# Effect of Fms-like tyrosine kinase 3 (FLT3) ligand (FL) on antitumor activity of gilteritinib, a FLT3 inhibitor, in mice xenografted with FL-overexpressing cells

**DOI:** 10.18632/oncotarget.27222

**Published:** 2019-10-22

**Authors:** Tatsuya Kawase, Taisuke Nakazawa, Tomohiro Eguchi, Hirofumi Tsuzuki, Yoko Ueno, Yasushi Amano, Tomoyuki Suzuki, Masamichi Mori, Taku Yoshida

**Affiliations:** ^1^ Drug Discovery Research, Astellas Pharma, Tsukuba-shi, Ibaraki, Japan

**Keywords:** FLT3, FL, FLT3 inhibitor, gilteritinib, antitumor activity

## Abstract

Therapeutic effects of FLT3 inhibitors have been reported in acute myeloid leukemia (AML) with constitutively activating *FLT3* mutations, including internal tandem duplication (ITD) and point mutation, which are found in approximately one-third of AML patients. One of the critical issues of treatment with FLT3 inhibitors in *FLT3*-mutated AML is drug resistance. FLT3 ligand (FL) represents a mechanism of resistance to FLT3 inhibitors, including quizartinib, midostaurin, and sorafenib, in AML cells harboring both wild-type and mutant *FLT3* (*FLT3*^wt^/*FLT3*^mut^). Here, we investigated the effect of FL on the efficacy of gilteritinib, a FLT3 inhibitor, in AML-derived cells *in vitro* and in mice. In contrast to other FLT3 inhibitors, FL stimulation had little effect on growth inhibition or apoptosis induction by gilteritinib. The antitumor activity of gilteritinib was also comparable between xenograft mouse models injected with FL-expressing and mock MOLM-13 cells. In the FLT3 signaling analyses, gilteritinib inhibited FLT3^wt^ and FLT3-ITD to a similar degree in HEK293 and Ba/F3 cells, and similarly suppressed FLT3 downstream signaling molecules (including ERK1/2 and STAT5) in both the presence and absence of FL in MOLM-13 cells. Co-crystal structure analysis showed that gilteritinib bound to the ATP-binding pocket of FLT3. These results suggest that gilteritinib has therapeutic potential in FLT3-mutated AML patients with FL overexpression.

## INTRODUCTION

Fms-like tyrosine kinase 3 (FLT3) is a member of the class III family of receptor tyrosine kinases expressed on early hematopoietic stem and progenitor cells, and plays an important role in hematopoiesis [1]. Wild-type FLT3 (FLT3^wt^) is activated by FLT3 ligand (FL), which promotes receptor dimerization, auto-phosphorylation, and cell proliferation via activation of its downstream signaling molecules, including STAT5, ERK1/2, and AKT [2]. FLT3 is made up of 5 functional domains: an immunoglobulin-like extracellular domain, a transmembrane domain, a juxtamembrane domain (JMD), a tyrosine kinase domain (TKD), and a small C-terminal domain [3]. Mutations at the JMD or TKD of *FLT3* induce constitutive kinase activation that is independent of FL, and occurs in approximately one-third of acute myeloid leukemia (AML) patients [4, 5]. In particular, in-frame duplications of 3 to >400 base pairs in the JMD, known as internal tandem duplications (ITDs), are the most common mutations, occurring in up to 30% of patients with AML, and are associated with poor prognosis [4–7]. Activating point mutations in the TKD are also observed in patients with AML, but at a lower frequency than ITD mutations [5, 8]. These activating mutations are oncogenic and render a state of “oncogene addiction” in this disease [5, 9–11]. Therefore, FLT3 is considered a promising drug target in AML patients with *FLT3* mutations.

A number of FLT3 inhibitors, including gilteritinib, midostaurin, quizartinib, and sorafenib, have been evaluated in clinical trials [12–15]. In 2017, the US Food and Drug Administration (FDA) and European Medicines Agency approved midostaurin for the treatment of adult patients with newly diagnosed AML with *FLT3* mutation in combination with standard chemotherapy [16]. Gilteritinib is a selective FLT3 inhibitor that inhibits both FLT3-ITD and FLT3-TKD mutations, and is classified as an ATP-competitive type I inhibitor [17]. Based on a phase 3 clinical trial, gilteritinib was recently approved by the Pharmaceuticals and Medical Devices Agency and FDA as monotherapy for patients with relapsed/refractory *FLT3*-mutated AML [18].

A number of resistance mechanisms to FLT3 inhibitors have been identified in preclinical and clinical studies, including acquired *FLT3* resistance mutations [19], other gene mutations such as *NRAS* [20], and altered protein expression such as that of FL [21], AXL kinase [22, 23], Pim kinase [24], or FGF2 [25]. In particular, one study reported that increased plasma concentrations of FL after chemotherapy induces resistance to FLT3 inhibitors—including midostaurin, quizartinib, sorafenib, and lestaurtinib—in AML cells with *FLT3*^wt^ or mutant *FLT3* (*FLT3*^mut^) [21]. The mechanism by which FL impedes the efficacy of FLT3 inhibitors is reportedly based on FL-dependent activation of FLT3^wt^ [26]. One study showed that the efficacy of quizartinib, a selective FLT3 inhibitor that binds to inactive forms of FLT3, was attenuated when cells co-expressing FLT3^wt/mut^ were stimulated with FL, resulting in activation of survival/growth signals via FLT3^wt^. Given that AML cells that harbor *FLT3* mutations co-express *FLT3*^wt^ [27], FL-dependent resistance to FLT3 inhibitors is considered one of the obstacles to treatment of AML patients with *FLT3*^wt/mut^. However, it remains unclear whether this effect is the same for all FLT3 inhibitors.

Here, we investigated whether the inhibitory effects of gilteritinib on tumor growth and FLT3 signal transduction are affected by FL in the human AML cell line MOLM-13, which harbors both *FLT3*^wt^ and *FLT3*-ITD. We subsequently generated xenograft mouse models using FL-expressing MOLM-13 cells to mimic the upregulated condition of soluble FL observed in AML patients treated with chemotherapy and FLT3 inhibitor, and evaluated the antitumor activity of gilteritinib. In addition, we investigated the inhibitory effects of gilteritinib on FLT3^wt^ and FLT3-ITD in HEK293 and Ba/F3 cells. Our results demonstrate that gilteritinib potently inhibits tumor growth even in the presence of FL due to comparable inhibitory efficacy against FLT3^wt^ and FLT3-ITD. These findings indicate that FL has no effect on the inhibitory effects of gilteritinib and suggest that gilteritinib has therapeutic potential in *FLT3*-mutated AML patients with FL overexpression.

## RESULTS

### Growth inhibition and apoptosis induction by gilteritinib with or without FL

**Figure 1:**
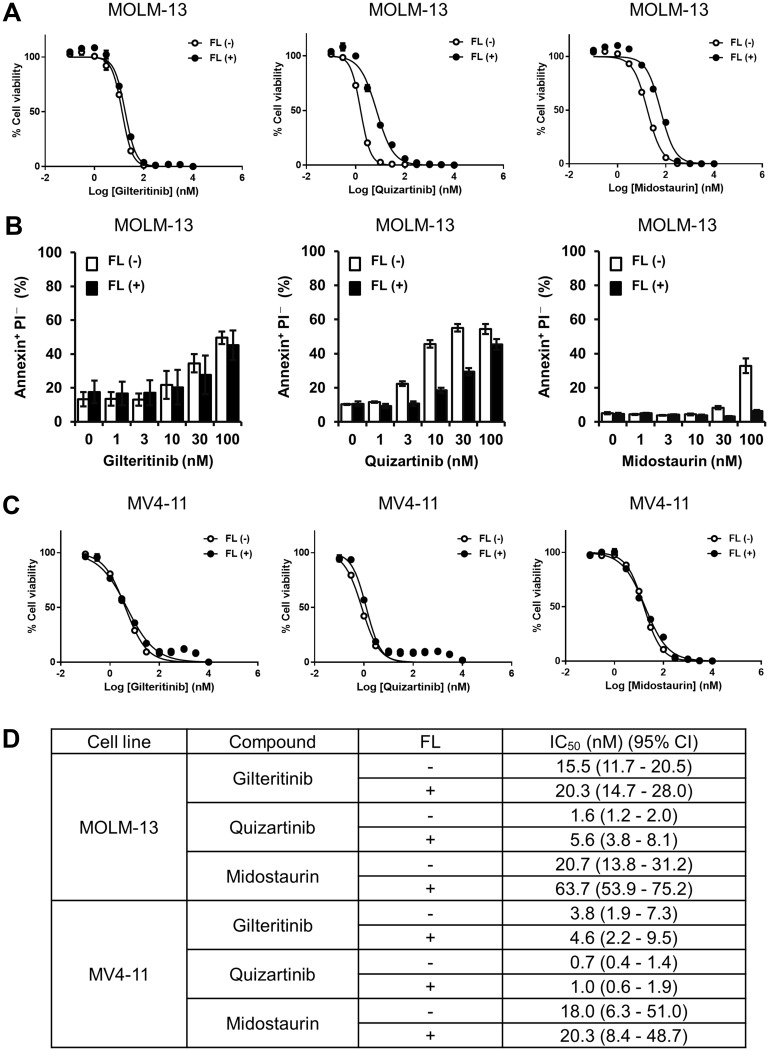
Growth inhibition and apoptosis induction by gilteritinib and quizartinib. (**A** and **C**) MOLM-13 and MV4-11 cells were treated with gilteritinib, quizartinib or midostaurin in the presence or absence of FL at 25 ng/mL for 3 days. Cell viability was measured using the CellTiter-Glo 2.0 Assay. At least 3 experiments were performed in duplicate or triplicate, and representative data are shown as mean ± SD. (**B**) MOLM-13 cells were treated with gilteritinib, quizartinib or midostaurin in the presence or absence of FL at 25 ng/mL for 2 days. Cells were harvested and stained with Annexin V and PI. Apoptotic cells were measured using flow cytometry. The experiment was performed in triplicate, and data are shown as mean ± SEM. (**D**) Cell viability analysis of MOLM-13 and MV4-11 cells treated with gilteritinib, quizartinib or midostaurin as in Figure 1A and 1C. Geometric mean IC_50_ values and 95% CIs were determined from at least 3 independent experiments. Abbreviations: CI, confidence interval; FL, FLT3 ligand.

To investigate whether the growth inhibitory effect of gilteritinib, a FLT3 inhibitor, is affected by FL stimulation, we treated MOLM-13 cells harboring both *FLT3*^wt^ and *FLT3*-ITD with gilteritinib in the presence or absence of FL. Given that FL stimulation reportedly impedes the effects of other FLT3 inhibitors [21, 26], we used quizartinib and midostaurin as reference compounds. Quizartinib inhibited the growth of MOLM-13 cells with IC_50_ values of 1.6 nM (95% CI: 1.2–2.0) and 5.6 nM (95% CI: 3.8–8.1) in the absence and presence of FL, respectively (Figure 1A and 1D). Midostaurin inhibited the growth of MOLM-13 cells with IC_50_ values of 20.7 nM (95% CI: 13.8–31.2) and 63.7 nM (95% CI: 53.9–75.2) in the absence and presence of FL, respectively (Figure 1A and 1D). Consistent with previous reports, our findings demonstrate that the growth inhibitory effects of quizartinib and midostaurin on MOLM-13 cells were attenuated in the presence compared with the absence of FL. In contrast, gilteritinib inhibited the growth of MOLM-13 cells with IC_50_ values of 15.5 nM (95% CI: 11.7–20.5) and 20.3 nM (95% CI: 14.7–28.0) in the absence and presence of FL, respectively, indicating that FL stimulation had little effect on the growth inhibitory effect of gilteritinib in MOLM-13 cells (Figure 1A and 1D).

In addition, we analyzed the ability of gilteritinib, quizartinib and midostaurin to induce apoptosis of MOLM-13 cells. We found that while FL stimulation had no effect on apoptosis induction by gilteritinib, the presence of FL attenuated apoptosis induction by quizartinib and midostaurin compared with the absence of FL (Figure 1B). Given that a previous study showed that FLT3^wt^ was responsible for inducing the FL-dependent attenuation of the growth inhibitory effect of quizartinib [26], we investigated the growth inhibitory effects of quizartinib and midostaurin using the human AML cell line MV4-11, which harbors a homozygous *FLT3*-ITD mutation. The inhibitory effects of quizartinib and midostaurin on the growth of MV4-11 cells were comparable between the presence and absence of FL (Figure 1C and 1D). These results indicate that unlike quizartinib and midostaurin, FL had no effect on the growth inhibitory effect of gilteritinib in AML cells with *FLT3*^wt^/*FLT3*-ITD.

### Antitumor activity of gilteritinib in xenograft mouse models

**Figure 2:**
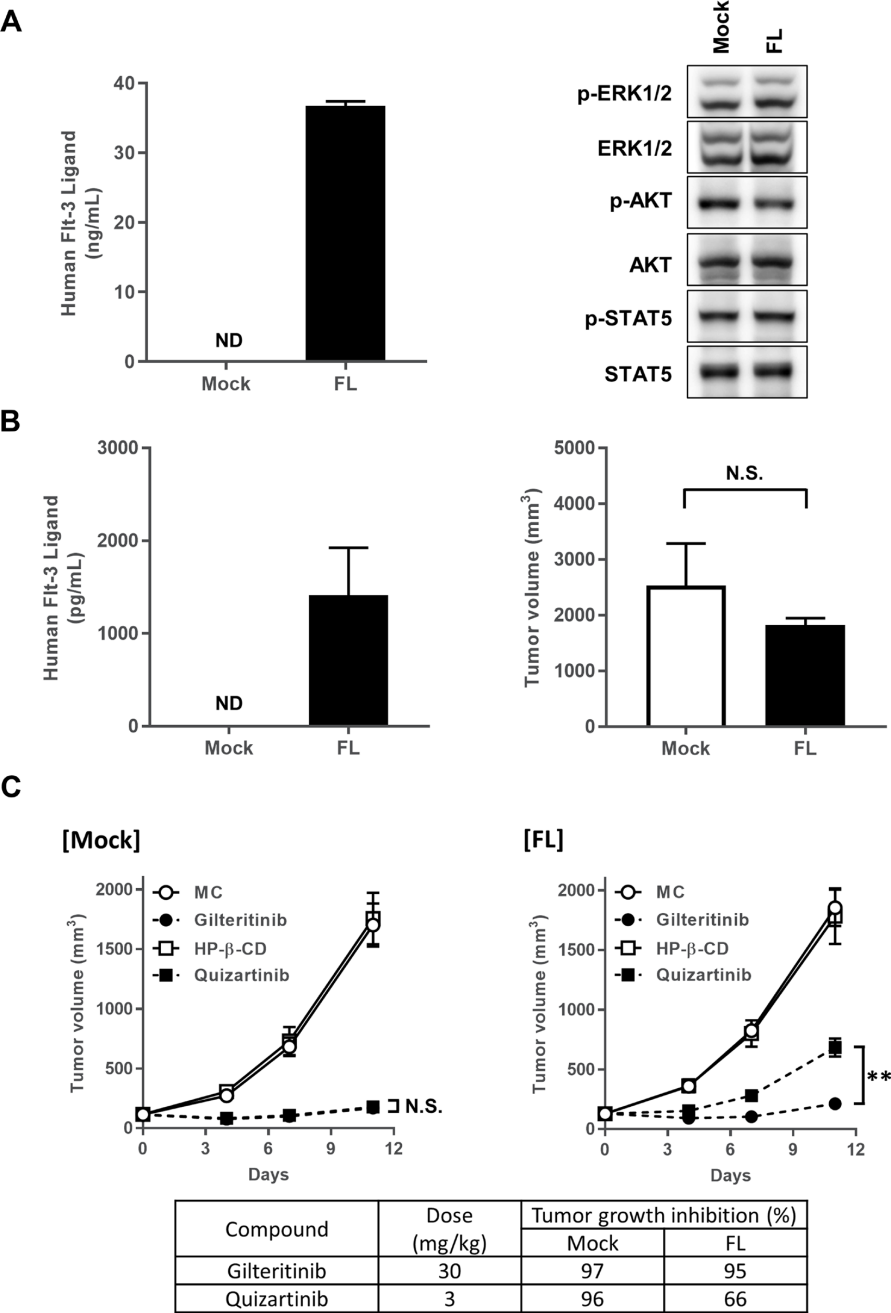
Antitumor efficacy of gilteritinib and quizartinib. (**A**) FL expression was detected in *FL*-expressing or mock MOLM-13 cells. *FL*-expressing or mock MOLM-13 cells were subjected to western blotting analysis using the indicated antibodies. (**B**) Plasma FL concentrations were determined on day 22, and data are shown as mean ± SEM (*n* = 5). Tumor volume was measured on day 18, and data are shown as mean ± SEM (*n* = 6). (**C**) Mice engrafted with FL-expressing or mock MOLM-13 cells were orally administered gilteritinib or quizartinib at 30 mg/kg or 3 mg/kg, respectively. Tumor volume was measured, and data are shown as mean ± SEM (*n* = 10). Tumor volume on day 11 was compared between the gilteritinib-treated group and quizartinib-treated group using Student’s *t*-test. ^**^ *P* < 0.01. Abbreviations: FL, FLT3 ligand; ND, not detected; N. S., not significantly different.

Given that FL had no effect on the growth inhibitory effect of gilteritinib *in vitro*, we next investigated the potential effect of FL on the antitumor activity of gilteritinib in xenograft mouse models. To generate a xenograft mouse model that mimics the upregulated condition of soluble FL observed in AML patients treated with chemotherapy or FLT3 inhibitor [21], we infected MOLM-13 cells with a retrovirus encoding a soluble FL or mock gene to generate FL-expressing or mock MOLM-13 cells, respectively. Analysis of the expression of FL and activities of FLT3 downstream molecules in FL-expressing and mock MOLM-13 cells demonstrated FL expression in the supernatant of FL-expressing cells but not mock cells. Further, the activities of the FLT3 downstream molecules were not significantly altered in FL-expressing cells compared to mock cells under the normal condition (Figure 2A). These cells were subcutaneously inoculated into the flank of mice and allowed to grow. Tumor volume and plasma FL concentrations were measured 18 and 22 days after inoculation, respectively. The growth rate of FL-expressing MOLM-13 tumors was not significantly different from that of mock MOLM-13 tumors. Further, plasma FL concentrations were detected in nude mice engrafted with FL-expressing cells but not mock cells (Figure 2B). Interestingly, plasma FL concentrations in FL-expressing tumor xenograft models on day 22 were similar to those observed in AML patients treated with chemotherapy and FLT3 inhibitor [21].

Next, we evaluated the antitumor activities of gilteritinib and quizartinib in these xenograft mouse models. Once-daily administration of gilteritinib at 30 mg/kg or quizartinib at 3 mg/kg per day for 11 days inhibited the growth of mock MOLM-13 tumors by 97% or 96%, respectively, indicating that the antitumor efficacies of gilteritinib (30 mg/kg) and quizartinib (3 mg/kg) in the mock-cell xenograft model were comparable (Figure 2C). When quizartinib (3 mg/kg) was administered to mice with FL-expressing MOLM-13 tumors, tumor growth was inhibited by 66%, indicating that the presence of FL attenuated the antitumor activity of quizartinib compared with that of gilteritinib (Figure 2C). As expected from our *in vitro* results, gilteritinib (30 mg/kg) showed similar efficacy to that for mock MOLM-13 tumors, inhibiting FL-expressing MOLM-13 tumor growth by 95% (Figure 2C). These results indicate that, unlike quizartinib, FL had no effect on the antitumor efficacy of gilteritinib *in vivo*.

### Inhibitory effects of gilteritinib on FLT3^wt^ and FLT3-ITD

We next investigated the mechanism of the lack of effect of FL on the antitumor activity of gilteritinib. Studies have reported that FL-induced activation of FLT3^wt^ is a key mechanism in FL-dependent resistance of FLT3 inhibitors: quizartinib has higher potency against FLT3-ITD than FLT3^wt^, which leads to FL-mediated impairment of antitumor activity by quizartinib [26, 28]. Therefore, we hypothesized that gilteritinib inhibits FLT3^wt^ with the same or greater efficacy than it inhibits FLT3-ITD.

**Figure 3:**
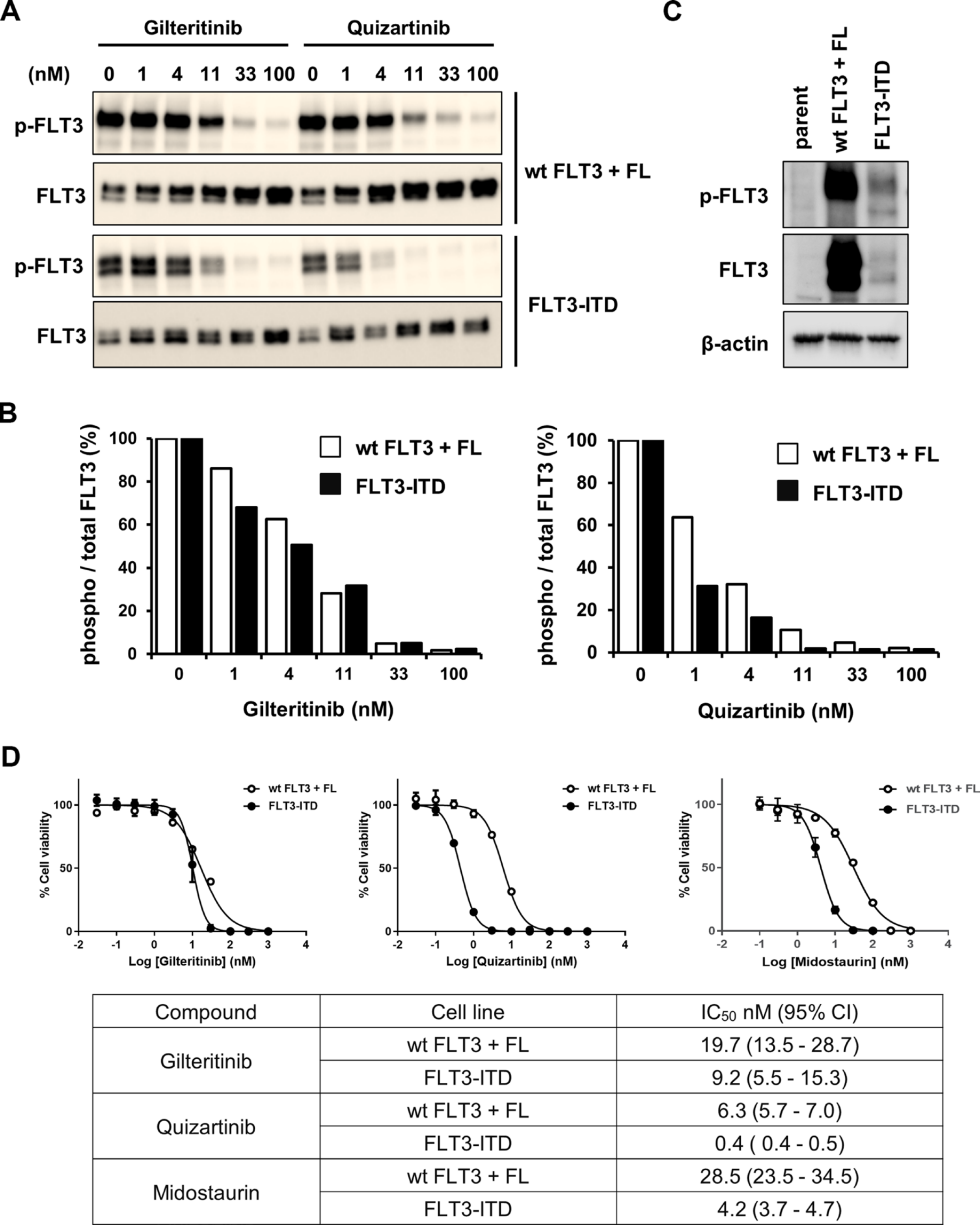
Inhibitory effects of gilteritinib and quizartinib on FLT3^wt^ and FLT3-ITD. (**A** and **B**) HEK293 cells were transfected with an *FLT3*^wt^- or *FLT3*-ITD-expressing vector. After culturing overnight, cells were treated with gilteritinib or quizartinib for 1 hour. FL (25 ng/mL) was added to *FLT3*^wt^-expressing cells simultaneously with the compounds. Cells were lysed and subjected to western blotting analysis using antibodies against total or phospho-FLT3 (Y591). The ratio of phospho/total FLT3 was calculated and is shown in the graph. (**C**) *FLT3*^wt^- or *FLT3*-ITD-expressing Ba/F3 cells and parental cells were subjected to western blotting analysis using antibodies against FLT3, phospho-FLT3 and β-actin. (**D**) *FLT3*^wt^- or *FLT3*-ITD-expressing Ba/F3 cells were treated with gilteritinib, quizartinib or midostaurin for 3 days. FL (25 ng/mL) was added to *FLT3*^wt^-expressing cells simultaneously with the compounds. Cell viability was measured using the CellTiter-Glo 2.0 Assay. Three experiments were performed in triplicate, and representative data are shown as the mean ± SD. Geometric mean IC_50_ values and 95% CIs were determined from 3 independent experiments. Abbreviations: CI, confidence interval; FL, FLT3 ligand; ITD, internal tandem duplication; wt, wild-type.

Because it is difficult to investigate the inhibitory effects of gilteritinib against FLT3^wt^ and FLT3-ITD in MOLM-13 cells, we instead examined the inhibitory effects of gilteritinib and quizartinib on auto-phosphorylation of the 2 types of FLT3 and on the cell proliferation of HEK293 and Ba/F3 cells. HEK293 cells were transfected with *FLT3*^wt^- or *FLT3*-ITD-expressing vectors and auto-phosphorylation levels of FLT3 were determined using western blotting. We found that while the inhibitory effects of gilteritinib on the auto-phosphorylation levels of FLT3^wt^ and FLT3-ITD were comparable, those of quizartinib were weaker on FLT3^wt^ compared with FLT3-ITD (Figure 3A and 3B). We subsequently generated *FLT3*^wt^- and *FLT3*-ITD-expressing Ba/F3 cells (Figure 3C) and investigated the inhibitory effects of gilteritinib and quizartinib on their growth. Gilteritinib inhibited the growth of *FLT3*^wt^- and *FLT3*-ITD-expressing Ba/F3 cells with IC_50_ values of 19.7 nM (95% CI: 13.5–28.7) and 9.2 nM (95% CI: 5.5–15.3), respectively (Figure 3D). In contrast, quizartinib inhibited the growth of *FLT3*^wt^- and *FLT3*-ITD-expressing Ba/F3 cells with IC_50_ values of 6.3 nM (95% CI: 5.7–7.0) and 0.4 nM (95% CI: 0.4–0.5), respectively (Figure 3D). In addition, midostaurin inhibited the growth of *FLT3*^wt^- and *FLT3*-ITD-expressing Ba/F3 cells with IC_50_ values of 28.5 nM (95% CI: 23.5–34.5) and 4.2 nM (95% CI: 3.7–4.7), respectively (Figure 3D). These results indicate that gilteritinib had similar inhibitory action on FLT3^wt^ and FLT3-ITD, while quizartinib and midostaurin had more potent inhibitory action on FLT3-ITD than on FLT3^wt^. The similar inhibitory potency against FLT3^wt^ and FLT3-ITD suggests that FL has no effect on the efficacy of gilteritinib.

### Inhibitory effects of gilteritinib on FLT3 downstream signaling molecules

**Figure 4:**
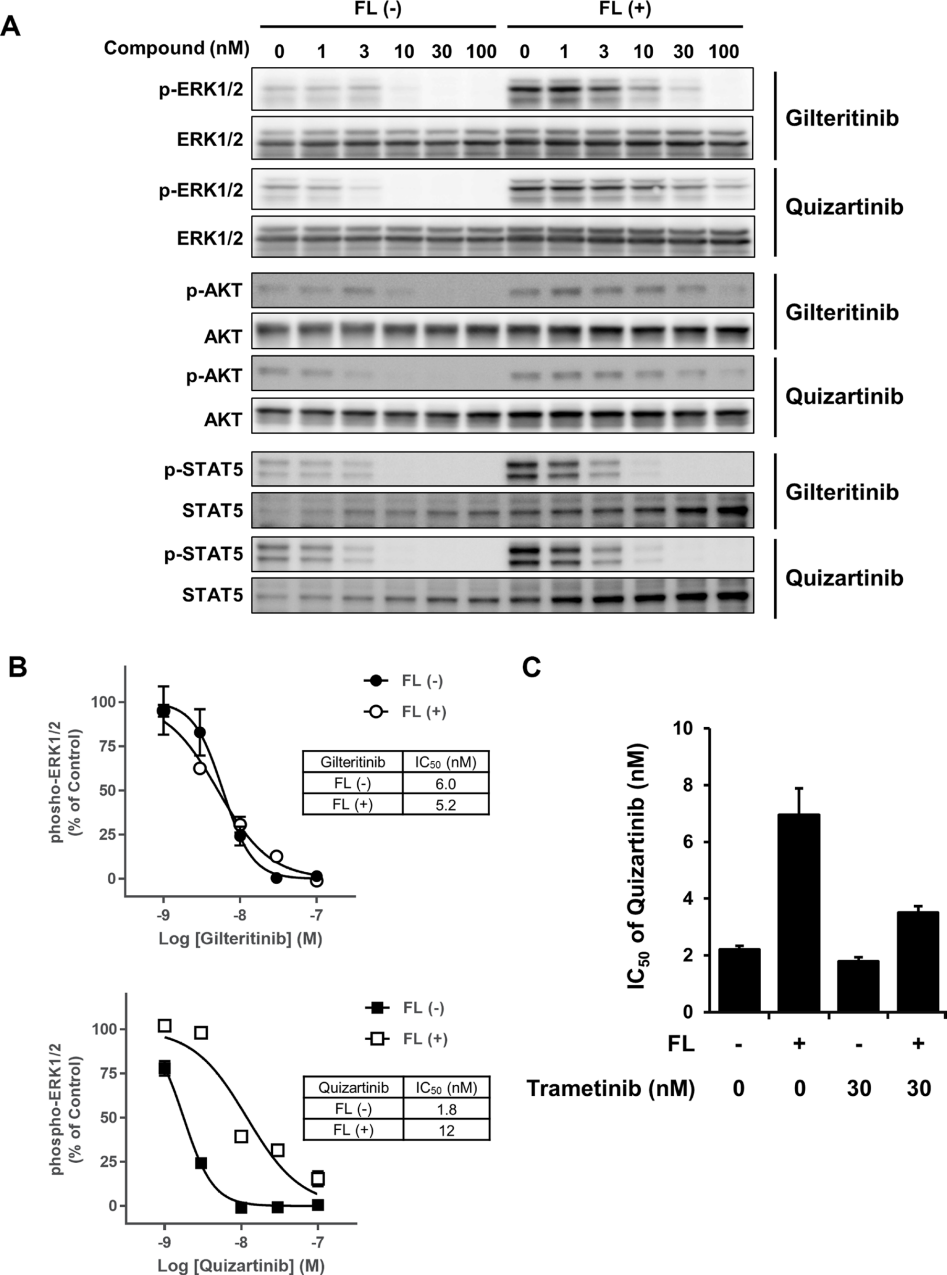
FLT3 downstream signaling analysis. (**A**) MOLM-13 cells were treated with gilteritinib or quizartinib in the presence or absence of FL at 25 ng/mL for 2 hours. Cells were lysed and subjected to western blotting analysis using the indicated antibodies. (**B**) Phospho-ERK1/2 levels were determined using the phospho-ERK assay kit. The experiment was performed in triplicate, and data are shown as mean ± SD. (**C**) MOLM-13 cells were treated with quizartinib in combination with or without trametinib in the presence or absence of FL at 25 ng/mL for 2 days. Cell viability was measured using the CellTiter-Glo 2.0 Assay. Cell viability was calculated by defining the survival of quizartinib-untreated cells with or without trametinib treatment and medium control wells as 100% and 0%, respectively. Three experiments were performed in triplicate, and data are shown as mean ± SEM. Abbreviation: FL, FLT3 ligand.

To investigate whether the presence or absence of FL affects the gilteritinib-mediated suppression of FLT3 downstream signaling molecules, we next evaluated the phosphorylation statuses of ERK1/2, STAT5, and AKT in MOLM-13 cells treated with gilteritinib or quizartinib. Treatment with gilteritinib and quizartinib without FL stimulation suppressed phosphorylation of all FLT3 downstream signaling molecules tested. In the presence of FL, quizartinib showed similar suppression of the phosphorylation of STAT5, but attenuated inhibitory efficacy for the phosphorylation of ERK1/2 and AKT (Figure 4A and Supplementary Figure 1). In contrast, in the presence of FL, gilteritinib showed similar suppression of the phosphorylation of ERK1/2 and STAT5, but not AKT (Figure 4A and Supplementary Figure 1).

Given that gilteritinib and quizartinib differed in their suppression of the phosphorylation of ERK1/2 in the presence of FL, we further investigated the effect of gilteritinib and quizartinib on the phosphorylation status of ERK1/2 in MOLM-13 cells in the presence or absence of FL using the advanced ERK phospho-T202/Y204 detection system. Similar to our initial findings, this assay showed that while the presence of FL attenuated the effects of quizartinib, it did not affect those of gilteritinib (Figure 4B). Given that Fangli et al reported that activation of the MAPK pathway plays a crucial role in FL-dependent resistance to FLT3 inhibitors in *FLT3*^wt^ and *FLT3*-ITD co-expressing 32D cells [26], we investigated whether trametinib, a potent and selective MEK inhibitor, recovers the efficacy of quizartinib in the presence of FL. We confirmed that treatment with trametinib to inhibit the MAPK pathway abolished the FL-dependent resistance to quizartinib (Figure 4C and Supplementary Figure 2). Therefore, these results indicate that the inhibitory effect of gilteritinib on the FLT3^wt^-MAPK pathway is crucial for overcoming FL-dependent resistance.

### Crystal structure of FLT3 in complex with gilteritinib

**Figure 5:**
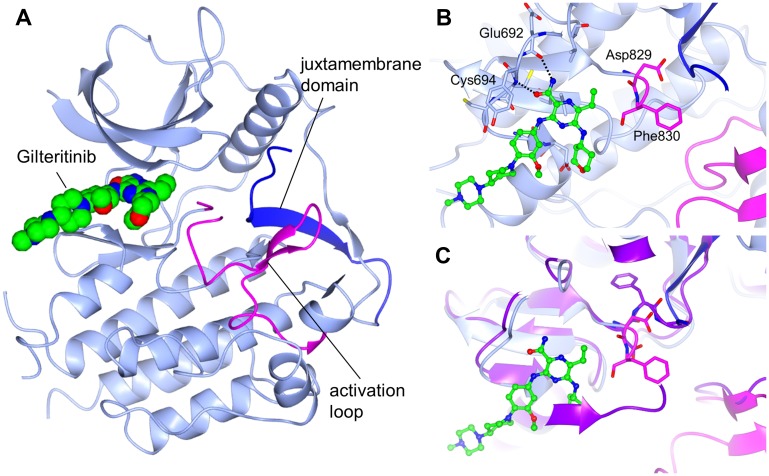
Crystal structure of FLT3 bound to gilteritinib. FLT3 is shown in light blue, the activation loop is shown in magenta, and the juxtamembrane domain is shown in blue. (**A**) Overall structure. (**B**) Binding mode of gilteritinib. Hydrogen bonds are shown as dashed lines. (**C**) Overlay with the c-Kit active structure (PDB 1PKG). c-Kit is shown in purple. DFG motifs are shown as a stick model.

To reveal the binding mode, we determined the crystal structure of the FLT3 kinase domain in complex with gilteritinib. As expected from the computational modeling performed in our previous report [17], gilteritinib bound to the ATP pocket of FLT3 (Figure 5A). While the JMD was clearly observed in the overall structure, a portion of the activation loop including Gly831 in the DFG motif was disordered. Asp829 and Phe830 in the DFG motif adopted the “DFG-out” conformation. In the binding mode of gilteritinib, two hydrogen bonds were formed between the carbamoyl group and the main chain atoms of Glu692 and Cys694 (Figure 5B). There was no distinct interaction between gilteritinib and the activation loop including Asp829 and Phe830.

## DISCUSSION

FLT3 is a promising molecular target in a subset of AML cases, and the therapeutic effects of FLT3 inhibitors have been reported in AML patients with *FLT3* mutations. One of the critical issues of treatment with FLT3 inhibitors in *FLT3*-mutated AML is drug-resistance. A number of resistance mechanisms against FLT3 inhibitors have been identified, including acquired resistance mutations of *FLT3*, other somatic mutations of cancer-related genes, and activation of alternative signaling pathways. Our previous report showed that gilteritinib has inhibitory activity against the TKD mutation and gate keeper mutation of FLT3, which confer resistance to quizartinib [17]. Here, we investigated the effect of increasing FL, another potential mechanism of resistance to FLT3 inhibitors, on the efficacy of gilteritinib.

First, we confirmed previous findings that FL attenuates the inhibitory effects of FLT3 inhibitors including midostaurin and quizartinib in *FLT3*-mutated AML cells (Figure 1). Surprisingly, and in contrast to the profile of other FLT3 inhibitors, our results demonstrated that FL had no effect on the growth inhibitory effect of gilteritinib. These findings were also confirmed in xenograft mouse models injected with FL-overexpressing AML cells.

Next, we investigated the underlying mechanism of the unique profile of gilteritinib. Previous report showed that the FL-mediated attenuation in the efficacy of quizartinib is a result of its weaker inhibitory effect on FLT3^wt^ compared with FLT3-ITD [26]. We therefore hypothesized that gilteritinib may show similar inhibitory potency against FLT3^wt^ and FLT3-ITD. Indeed, in the analyses of FLT3 signaling, gilteritinib inhibited FLT3^wt^ and FLT3-ITD to a similar extent in HEK293 and Ba/F3 cells, and also similarly suppressed FLT3 downstream signaling molecules, including ERK1/2 and STAT5, even in the presence of FL in MOLM-13 cells.

These findings may be clinically meaningful for several reasons. First, an FL increase has been observed in AML patients treated with a FLT3 inhibitor or chemotherapy [21]. The plasma concentrations of FL in the xenograft model established by inoculating FL-expressing MOLM-13 cells in our study were similar to those in AML patients [21]. In addition, detectable phosphorylation of FLT3^wt^, but not FLT3-ITD, has been observed using the plasma inhibitory activity assay with human plasma samples from patients administered quizartinib [28], suggesting that the efficacy of quizartinib may also be attenuated in the presence of FL in AML patients. Furthermore, single cell analysis of AML blasts in patients has revealed the presence of AML cells with *FLT3*-ITD co-expressing *FLT3*^wt^ [27]. Therefore, the FL increase may be correlated with resistance to treatment with FLT3 inhibitors. Our results suggest that gilteritinib may overcome the FL increase and lengthen the duration of efficacy, although further research is needed to confirm this in patients. In this study, we investigated the inhibitory activity of gilteritinib using the ITD sequence derived from MOLM-13 cells. A number of ITD mutations have been observed in AML patients with *FLT3*-ITD due to the presence of different ITD insertion sequences. Therefore, future studies should examine the efficacy of gilteritinib in the presence of FL using primary AML cells with various ITD mutations.

Tyrosine kinase inhibitors are classified into 2 types based on their affinity to receptors. Type I inhibitors such as gilteritinib, midostaurin, and lestaurtinib bind to both the active and inactive forms of FLT3, while type II inhibitors such as quizartinib bind only to inactive FLT3. However, given that both type I and II inhibitors are affected by FL—with the exception of gilteritinib—we propose that selectivity against FLT3^wt^ and FLT3-ITD may be more important than the inhibitors’ classification type.

The crystal structure of the complex formed between FLT3 and gilteritinib was obtained using the kinase domain of FLT3^wt^, including the JMD, which has been reported to bind to the active site of FLT3 and to stabilize the autoinhibited form [29]. In the complex structure with gilteritinib, the JMD bound to the active site, indicating that FLT3 adopted the autoinhibited form. Given that gilteritinib did not interact with the activation loop or the JMD, gilteritinib may also bind to the active form of FLT3, including the constitutively active form of FLT3-ITD. To elucidate whether gilteritinib binds to the active form, the c-Kit active structure [30] was superimposed onto the FLT3 complex structure with gilteritinib (Figure 5C). The activation loop of FLT3 was predicted to adopt a similar conformation to the c-Kit activation loop because the amino acid residues are well conserved. Gilteritinib was far from the active conformation of the activation loop, suggesting that it likely binds to the active form of FLT3 as well as the autoinhibited form. These findings correspond to the similar inhibitory activity of gilteritinib against FLT3^wt^ and FLT3-ITD.

FL is reportedly involved in promoting the differentiation of hematopoietic progenitor cells into dendritic cells (DC) [31]. A potential downside of the inhibitory effect of gilteritinib on FLT3^wt^ is that it may affect DC differentiation and immune function. However, gilteritinib has inhibitory activity against AXL, which has cancer immunosuppressive activity [17, 32]. The mechanism by which gilteritinib affects cancer immunity is currently unknown.

Our results suggest that gilteritinib has therapeutic potential in *FLT3*-mutated AML patients with FL overexpression. In addition, we propose that gilteritinib may be a good combination drug with chemotherapies that increase plasma concentrations of FL in *FLT3*-mutated AML patients and/or an effective monotherapy for AML patients with FL upregulation after chemotherapy.

## MATERIALS AND METHODS

### Regents and antibodies

Gilteritinib hemifumarate (gilteritinib) and quizartinib dihydrochloride were synthesized by Astellas Pharma, Inc. and Sumika Technoservice Co., respectively. Quizartinib and midostaurin were purchased from Sellek Chemicals and LC Laboratories, respectively. Trametinib was synthesized by OSI Pharma, Inc. Gilteritinib hemifumarate was dissolved in dimethyl sulfoxide (DMSO) or suspended in 0.5% methylcellulose (MC) for *in vitro* or *in vivo* experiments, respectively. Quizartinib, midostaurin, and trametinib were dissolved in DMSO for *in vitro* experiments. Quizartinib dihydrochloride was dissolved in 22% 2-hydroxypropyl-β-cyclodextrin (HP-β-CD) for *in vivo* experiments. Recombinant human FLT3 ligand protein was purchased from R&D Systems, Inc. Matrigel was purchased from BD Biosciences. The following antibodies were used for immunoblotting: anti-phospho-Stat5 (Y694) (BD biosciences), anti-FLT3, anti-β-actin, anti-p44/42 MAPK (Erk1/2), anti-Akt, anti-Stat5, anti-phospho-FLT3 (Y591), anti-phospho-p44/43 MAPK (Erk1/2) (T202/Y204), and anti-phospho-Akt (S473) (Cell Signaling Technology).

### Plasmids

Human *FLT3*^wt^ (NM_004119) and *FLT3*-ITD were N-terminally tagged with flag and cloned into the pMXs-Puro retroviral vector (Cell Biolabs). The ITD sequence of *FLT3*-ITD was derived from MOLM-13 cells [33]. *FLT3LG* (a. a. 1-181, NM_001204502) encoding the soluble form of FL was cloned into the pMXs-Puro retroviral vector.

### Cell lines, cell culture, and stable cell lines

MOLM-13 cells were purchased from the German Collection of Microorganisms and Cell Cultures; MV4-11 and HEK293 cells from the American Type Culture Collection; retroviral packaging cell line GP2-293 from Clontech; and Ba/F3 cells from RIKEN Cell bank. MOLM-13 cells were cultured in Roswell Park Memorial Institute (RPMI)-1640 (Sigma-Aldrich) medium supplemented with 10% fetal bovine serum (FBS) and 1% penicillin-streptomycin (PS); MV4-11 in Iscove’s Modified Dulbecco’s Medium (IMDM; Thermo Fisher Scientific) supplemented with 10% FBS and 1% PS; HEK293 and GP2-293 cells in Dulbecco’s Modified Eagle’s Medium (DMEM; Sigma-Aldrich) supplemented with 10% FBS and 1% PS; and Ba/F3 cells in RPMI-1640 supplemented with 10% FBS, 1% PS, and 10 ng/mL mouse interleukin (mIL)-3 (R&D systems).

FL-expressing or mock MOLM-13 cells were generated by Astellas Pharma, Inc. The pMXs-Puro retroviral vector containing the human soluble *FLT3LG* gene or empty vector was co-transfected with pVSV-G (Clontech) into GP2-293 cells using lipofectamine 2000 (Invitrogen) to produce the virus. MOLM-13 cells were then infected with the viral supernatant containing the *FLT3LG* gene or empty vector. Stable transfectants were obtained and maintained under selection pressure using puromycin (Nacalai Tesque) at 1.0 μg/mL. *FLT3*^wt^- or *FLT3*-ITD-expressing Ba/F3 cells were also generated by Astellas Pharma, Inc. The pMXs-Puro retroviral vector containing the *FLT3*^wt^- or *FLT3*-ITD gene was transfected into Platinum-E cells using lipofectamine 2000 to produce the virus. Ba/F3 cells were then infected with viral supernatant containing each *FLT3* gene. *FLT3*^wt^- or *FLT3*-ITD-expressing Ba/F3 cells were selected using puromycin at 1.0 μg/mL and subsequently cultured in mIL-3-deprived medium with or without 10 ng/mL FL, respectively.

### Cell viability assay

MOLM-13 and MV4-11 cells were seeded at 10,000 cells/well in 96-well plates. After culturing overnight, cells were treated with gilteritinib, quizartinib, or midostaurin in the presence or absence of FL at 25 ng/mL for 3 days [21, 26]. *FLT3*^wt^- or *FLT3*-ITD-expressing Ba/F3 cells were seeded at 1,000 cells/well in 96-well plates and treated with gilteritinib or quizartinib for 3 days in the presence or absence of FL at 25 ng/mL for 3 days. Cell viability was measured using the CellTiter-Glo 2.0 Assay (Promega). Measurement of luciferase activity was performed using the ARVO X3 (PerkinElmer) or SpectraMax Paradigm (Molecular Devices). Data were analyzed using GraphPad PRISM 7 software (GraphPad). Cell viability was calculated by defining the survival of untreated cells and medium control wells as 100% and 0%, respectively.

### Apoptosis assay

MOLM-13 cells were seeded at 2 × 10^5^ cells/well in 12-well plates and treated with gilteritinib, quizartinib or midostaurin in the presence or absence of FL at 25 ng/mL for 2 days. Cells were harvested and stained with Annexin V and PI using the FITC Annexin V Apoptosis Detection Kit I (BD Biosciences). Apoptotic cells were analyzed using MACS Quant Analyzer10 (Miltenyi Biotec). Flow cytometric data were analyzed using FlowJo software (FlowJo).

### Animal models for *in vivo* studies

All experimental animal procedures were approved by the Institutional Animal Care and Use Committee of Astellas Pharma, Inc. The Tsukuba Research Center of Astellas Pharma, Inc. has been awarded accreditation status by AAALAC International. Mice were maintained on water and a standard diet throughout the experimental procedures. Four-week-old male nude mice (BALB/c nu/nu) were purchased from Charles River Laboratories Japan, Inc.

### *In vitro* FL detection

FL-expressing or mock MOLM-13 cells were seeded at 3 × 10^5^ cells/well in 6-well plates. After culturing for 4 days, the supernatant of both cell types was collected and the amount of FL was quantified using the Human Flt-3 Ligand Quantikine ELISA Kit (R&D Systems).

### *In vivo* xenograft studies for FL detection

FL-expressing or mock MOLM-13 cells were subcutaneously inoculated into the flank of mice at 5 × 10^6^ cells/0.1 mL (matrigel: PBS = 1:1)/mouse and allowed to grow. Blood samples were collected 22 days after inoculation and the amount of FL in mouse plasma samples was quantified using the Human Flt-3 Ligand Quantikine ELISA Kit.

### *In vivo* xenograft studies on the antitumor activity of gilteritinib and quizartinib

FL-expressing or mock MOLM-13 cells were subcutaneously inoculated into the flank of mice at 5 × 10^6^ cells/0.1 mL (matrigel: PBS = 1:1)/mouse and allowed to grow. Seven days after inoculation, the mice were divided into 4 groups (gilteritinib, quizartinib, vehicle for gilteritinib, and vehicle for quizartinib; *n* = 10 each) such that the mean tumor volume was similar among the groups on the first day of administration (day 0). Gilteritinib (30 mg/kg) or quizartinib (3 mg/kg) was orally administered once daily to these mice for 11 days. The dose of each compound was expressed as the salt form. Tumor diameter was measured using a caliper on days 0, 4, 7, and 11, and tumor volume was determined by calculating the volume of an ellipsoid using the formula: length × width^2^ × 0.5. Body weight was measured using a standard balance.

### Statistical analysis

For the *in vivo* subcutaneous xenograft mouse model, values are expressed as mean ± SEM. Tumor volume in the gilteritinib-treated group was compared with that in the quizartinib-treated group using Student’s *t* test. *P* < 0.05 was considered statistically significant. Microsoft Excel (Microsoft) and GraphPad Prism (GraphPad Software) were used for data processing.

### Western blotting analysis

Cells were lysed with cell lysis buffer (Cell Signaling Technology) or RIPA buffer (Thermo Fisher Scientific) supplemented with Halt Protease & Phosphatase Inhibitor Cocktail (Thermo Fisher Scientific). Protein concentrations were determined using the BCA Protein Assay Kit (Thermo Fisher Scientific). Equal amounts of total protein were resolved by SDS-PAGE and transferred to PVDF membranes. After blocking at room temperature with Blocking One (Nacalai Tesque) or PVDF Blocking Reagent for Can Get Signal (TOYOBO Life Science), the membranes were incubated overnight with primary antibodies. After washing with TBS-T, membranes were incubated with horseradish peroxidase-conjugated secondary antibody for 1 hour at room temperature. Proteins of interest were visualized by enhanced chemiluminescence using the ECL Prime Western Blotting Detection System (GE Healthcare), Western Lightning Plus-ECL (PerkinElmer), or Western Lightning Ultra (PerkinElmer), and detected with LAS-4000 (GE Healthcare) or LAS-4000EPUVmini (Fujifilm) and quantified using ImageQuant software (GE Healthcare) or Multi Gauge software (Fujifilm).

### Quantification of phospho-ERK1/2

MOLM-13 cells were seeded at 3 ×10^6^ cells/well in 6-well plates and treated with gilteritinib or quizartinib in the presence or absence of FL at 25 ng/mL for 2 hours. Cells were harvested and phospho-ERK levels were evaluated using the Advanced ERK phospho-T202 /Y204 kit (Cisbio) and EnVision (Perkin Elmer).

### X-ray structural analysis

X-ray diffraction data were collected from crystals of the complex formed between human FLT3 and gilteritinib using the PXII/X10SA beamline at the Swiss Light Source (Paul Scherrer Institut, Villigen, Switzerland) under cryogenic conditions. Data were processed using XDS and XSCALE software [34]. A previously solved structure of hFLT3 was used as the search model. Subsequent model building and refinement was performed according to standard protocols using CCP4 [35] and COOT [36] software packages. Refinement using the translation/libration/screw-rotation (TLS) model (using REFMAC5 [37], CCP4) was performed. Ligand parameters and corresponding library files were generated using CORINA (Molecular Networks GmbH, Nürnberg, Germany). The water model was built using the COOT “Find waters” algorithm by placing water molecules at peaks of the Fo-Fc map contoured at 3.0 with subsequent refinement using REFMAC5, and checking all waters using the COOT validation tool. The coordinates were deposited into the Protein Data Bank (accession code 6JQR). All crystallographic work was performed by Proteros Biostructures GmbH (Martinsried, Germany).

## SUPPLEMENTARY MATERIALS


