# Supplementary material for: Effect of Fms-like tyrosine kinase 3 (FLT3) ligand (FL) on antitumor activity of gilteritinib, a FLT3 inhibitor, in mice xenografted with FL-overexpressing cells
Source: Oncotarget. 2019 Oct 22;10(58):6111–23. doi: 10.18632/oncotarget.27222 (PMC6817455; doi:10.18632/oncotarget.27222)
Supplement: Supplementary file 1 [file oncotarget-10-6111-s001.pdf]

Effect of Fms-like tyrosine kinase 3 (FLT3) ligand (FL) on antitumor activity of gilteritinib, a FLT3 inhibitor, in mice xenografted with FL-overexpressing cells

SUPPLEMENTARY MATERIALS

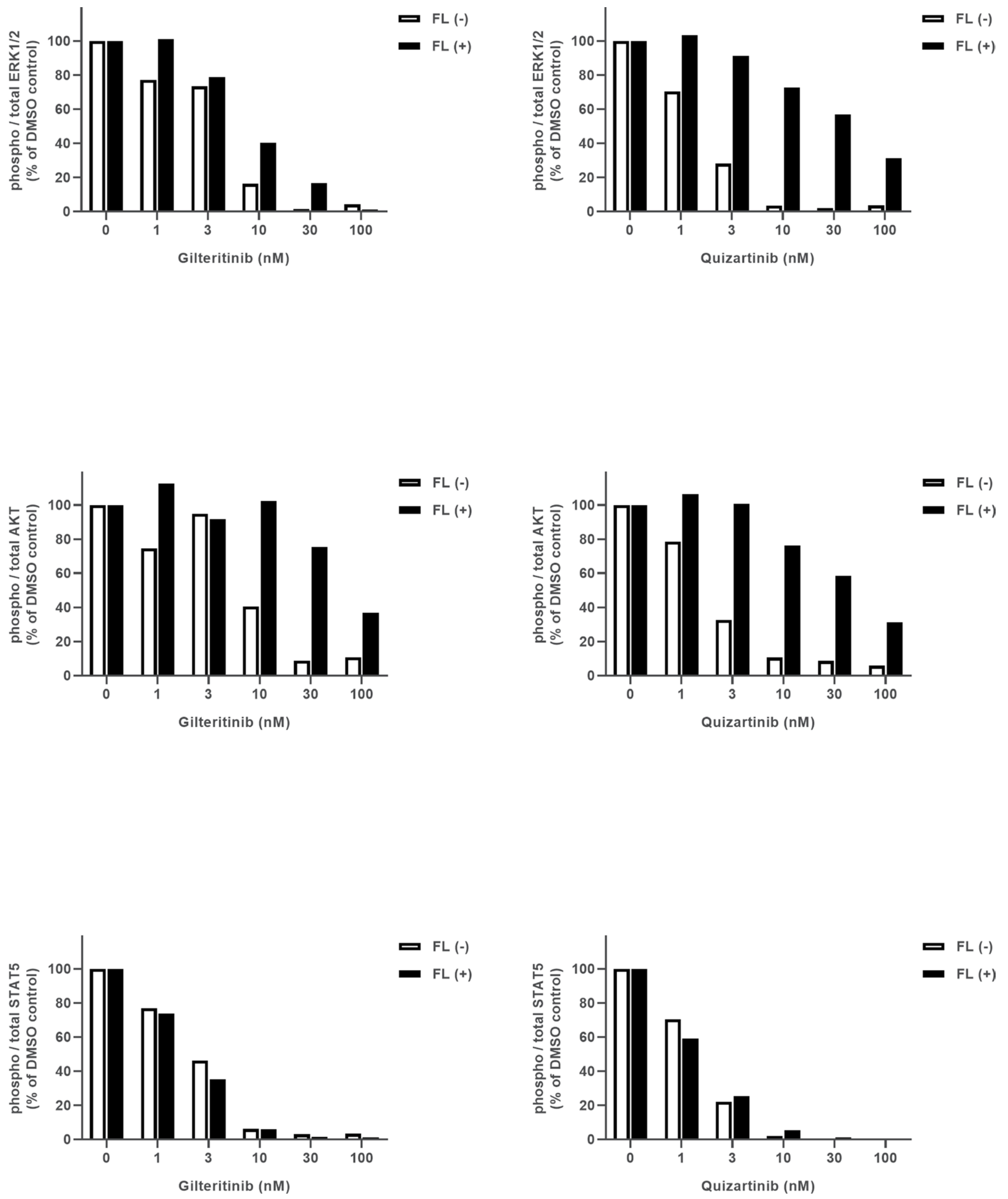

Supplementary Figure 1: The ratios of phospho/total ERK1/2, AKT and STAT5 were calculated from the results shown in Figure 4A and are shown in the graph.

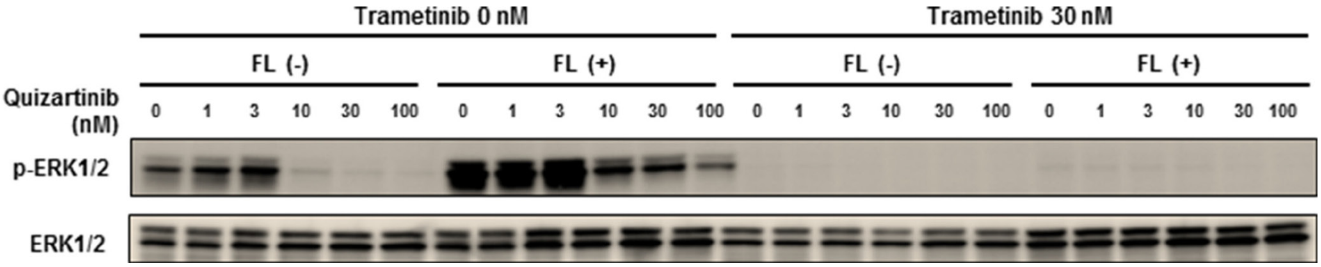

Supplementary Figure 2: Western blotting analysis of phospho-ERK1/2 and ERK1/2. MOLM-13 cells were treated with quizartinib in combination with or without trametinib in the presence or absence of FL at 25 ng/mL for 24 hours. Cells were lysed and subjected to western blotting analysis using the indicated antibodies. Abbreviation: FL, FLT3 ligand.
